# Prescription of potentially inappropriate medication to older patients presenting to the emergency department: a nationally representative population study

**DOI:** 10.1038/s41598-018-30184-4

**Published:** 2018-08-06

**Authors:** Chirn-Bin Chang, Hsiu-Yun Lai, Shinn-Jang Hwang, Shu-Yu Yang, Ru-Shu Wu, Hsing-Cheng Liu, Ding-Cheng Chan

**Affiliations:** 1 Department of Internal Medicine, National Taiwan University Chu-Tung Branch, Hsin-Chu County, Taiwan; 2 Department of Geriatrics and Gerontology, National Taiwan University Hospital, Taipei, Taiwan; 3 Department of Internal Medicine, National Taiwan University Hospital, Taipei, Taiwan; 4 Department of Family Medicine, National Taiwan University Hospital Hsin-Chu Branch, Hsin-Chu City, Taiwan; 5 Department of Family Medicine, Taipei Veteran General Hospital, Taipei, Taiwan; 6 National Yang-Ming University, School of Medicine, Taipei, Taiwan; 7 College of Pharmacy, Kaohsiung Medical University, Kaohsiung, Taiwan; 8 Taipei City Psychiatry Center, Taipei City Hospital, Taipei, Taiwan; 9 Department of Pharmacy, National Taiwan University Hospital, Taipei, Taiwan; 10 Department of Psychiatry, School of Medicine, Taipei Medical University, Taipei, Taiwan; 11 Superintedent Office, National Taiwan University Hospital Chu-Tung Branch, Hsin-Chu County, Taiwan

## Abstract

Potentially inappropriate medication (PIM) was associated with adverse clinical outcomes and higher healthcare resource utilization among older patients. In order to investigate the prevalence of PIM use based on three different sets of criteria and their associated factors among older patients in the emergency department (ED) in Taiwan. The National Health Insurance Research Database was used for this cross-sectional study. Older patients who visited the ED at least once in 2009 were enrolled. PIMs were identified based on the Beers Criteria, PIM-Taiwan criteria, and PRISCUS criteria. Average patient age was 76.7 ± 7.4 years and patients visited the ED 1.8 ± 2.1 times in 2009. The prevalence and frequency of being prescribed at least one PIM at each visit were high according to all three sets of criteria. Performance of the PIM-Taiwan criteria was only inferior to that of the Beers Criteria. The most important factor associated with PIM was the number of medications prescribed in the ED, and PIM use was associated with higher annual health resource utilization in the ED. PIM use was a significant issue and was associated with higher annual emergency care resource utilization in the ED.

## Introduction

Emergency department (ED) utilization is increasing for older adults and the number of annual visits is rapidly increasing^1,2^. In Taiwan, patients can visit the ED of private or public hospitals without a referral^3^. Older patients have more chronic conditions with potential acute exacerbations than do younger patients. In previous studies, older adults visited the ED more frequently compared to their relative proportion of overall Taiwanese beneficiaries^2^. They also underwent more laboratory and imaging studies, were prescribed more medications, and benefited from more staff care time compared with younger patients^2^. Among older adults, ED visits can be regarded as sentinel events for declining health status and a higher risk of adverse outcomes^1^. Therefore, physicians in the ED need to provide complex care and prevent iatrogenic diseases for older ED patients.

Adverse drug events (ADEs) account for the majority of iatrogenic complications of pharmacotherapy. In a previous report, the majority of these events were preventable^4^ and had been important targets for improvements to healthcare quality. To prevent ADE, prescriptions for older adults should consider the influence of the aging processes and chronic diseases on pharmacodynamics and pharmacokinetics. In addition, some medications have a higher risk of adverse events than efficacy rates. Potentially inappropriate medication (PIM) lists and criteria have been developed to prevent the use of this high-risk medication among the older population. The Beers Criteria^5^ have been widely used for PIM-related studies but are likely not applicable to all countries. Variations in available medications and prescribing preferences in different countries has led to the establishment of country-specific PIM lists, including the PIM-Taiwan criteria (Taiwan) and PRISCUS (German) criteria^6,7^. In previous studies, PIM was associated with adverse clinical outcomes and higher healthcare resource utilization^8–10^. Decreasing the rate of PIM prescription is a strategy that can be used to reduce the incidence of ADE among the older population.

The prevalence of PIM prescription among older ED patients increased from 5.6% to 26%^11–16^ with older Beers Criteria. Prescribing higher numbers of medications in the ED increases the risk of PIM use, and PIM use in the ED has been associated with ED attendance. To avoid further ADEs upon discharge from the ED, avoiding the prescription of PIMs in the ED has been recommended^17^. To date, a limited number of studies using the most updated (2015) Beers Criteria and country-specific PIM criteria^12–16,18–20^ have been applied to determine the prevalence of PIM and associated factors among older ED patients. Understanding this important issue in the ED will highlight concerns regarding PIM use and could lead to changes in prescribing preferences among ED physicians. However, only the Beers criteria had been applied to investigate the impacts of PIM among older ED patients in Taiwan. PIM-Taiwan had been published in 2012^6^ but this country-specific PIM criteria have not been applied to this population.

The primary aim of this study was to determine the frequency of administration of PIMs and common PIM-related diagnoses based on country-specific criteria (PIM-Taiwan criteria) and non-country specific criteria (2015 Beer criteria and PRISCUS criteria) among older ED patients by conducting a cross-sectional study based on Taiwan National Health Insurance Research Database. The secondary aim was to determine which patient- and ED visit-level factors were associated with PIM prescription.

## Results

### Characteristics of older adult patients, physicians, hospitals, and ED visits

In 2009, the NHIRD data captured a total of 313,733 older adults who had visited the ED at least once. Patient, physician, and hospital basic characteristics are presented in Table 1. The study population had an average age of 76.7 ± 7.4 years and an equal gender distribution. On average, patients had 1.8 ± 2.1 ED visits in 2009 and nearly 30% of visits were to EDs located in medical centers. Fifty-seven percent of physicians who treated older ED patients specialized in emergency medicine.

**Table 1:** Characteristics of older adult patients, physicians, hospitals, and ED visits in 2009.

Characteristics	Number (%) or mean (SD)
**Patient characteristics (N** = **313,733)**	
Male	159,452 (50.8)
Female	154,281 (49.2)
Age (years)	76.7 ± 7.4
Age group (years) 65–74	139,490 (44.5)
75–84	128,668 (41.0)
≥85	45,575 (14.5)
No. of ED visits/patient/year	1.8 ± 2.1
No. of hospitals/patient/year	1.2 ± 0.5
No. of physicians/patient/year	1.7 ± 1.3
No. of diagnoses/patient/year	3.0 ± 2.2
No. of medications/patient/year	6.2 ± 4.9
<4 medications/patient/year	100,297 (31.97)
4–6 medications/patient/year	108,495 (34.58)
>6 medications/patient/year	104,941 (33.45)
**Physician characteristics**	
Male	537,972 (93.1)
Female	39,826 (6.9)
Age (years)	40.9 ± 8.2
≤40	299,602 (51.7)
>40	280,194 (48.3)
Physicians specializing in emergency medicine	294,012 (56.9)
**Hospital characteristics (Level of accreditation)**	
Academic medical center	163,476 (28.2)
Non-medical center	416,320 (71.8)
**Visit characteristics (N** = **579,796)**	
No. of medications/visit/year	4.1 ± 2.4
Duration of medication/visit/year (day)	2.4 ± 1.7
Cost of medication/visit/year (TWD)	345.2 ± 2160.7

ED: emergency department; SD: standard deviation; TWD: New Taiwan Dollar, equivalent to 0.03 USD at time of publication.

### Characteristics of three sets of explicit criteria for PIM use and leading ten PIMs in the emergency department

The prevalence of PIM use and frequency of visits associated with PIMs differed between the three sets of PIM criteria used. Among the three sets of criteria, the 2015 version of the Beers Criteria included a higher number of statements and medications (Table 2). The proportion of patients administered at least one PIM (63.7%) and frequency of visits at which at least one PIM was prescribed (53.4%) were also highest according to the 2015 version of the Beers Criteria and lowest according to the PRISCUS criteria. Among PIM user, on average one PIM was prescribed in the ED for each patient in 2009 and nearly every visit to the ED was associated with at least one PIM in the Beers Criteria. The top ten PIMs were listed, indicating the most common diagnosis made for prescribing each medication. Ketorolac ranked first in the Beers Criteria and PIM-Taiwan criteria, whereas diphenhydramine ranked first in the PRISCUS criteria (Table 3). The top three leading diagnoses for PIM use in the ED were fever, dizziness and giddiness, and abdominal pain.

**Table 2:** Characteristics of three sets of explicit criteria for PIM use.

	Beers Criteria	PIM-Taiwan criteria	PRISCUS criteria
Year established	2015	2012	2010
Country	USA	Taiwan	Germany
No. of statements	36	24	15
No. of medications	151	84	83
Patients exposed to ≥1 PIM, n (%)	199,882 (63.7)	132,186 (42.1)	101,646 (32.4)
No. of PIMs per patient (mean ± SD)	1 ± 1.5	0.6 ± 1.2	0.5 ± 1.1
Visits with ≥1 PIM, n (%)	309,800 (53.4)	186,673 (32.2)	141,576 (24.4)
No. of PIMs per visit (mean ± SD)	0.9 ± 1.1	0.4 ± 0.7	0.3 ± 0.7

PIM: potentially inappropriate medication.

**Table 3:** Leading ten PIMs (N = 2,728,962) and associated primary diagnosis in the emergency department.

Beers Criteria (493, 216, 18.1%)			Taiwan-PIM criteria (248, 517, 9.1%)			PRISCUS criteria (207, 865, 7.6%)		
PIM item	N (%)	Most common Dx	PIM items	N (%)	Most common Dx	PIM items	N (%)	Most common Dx
Ketorolac (M01AB15)	72,834 (14.8)	Fever (780.6)	Ketorolac (M01AB15)	72,834 (29.3)	Fever (780.6)	Diphenhydramine (R06AA02)	48,911 (23.5)	Dizziness and giddiness (780.4)
Metoclopramide (A03FA01)	70,525 (14.3)	Abdominal pain (789.00)	Diphenhydramine (R06AA02)	48,911 (19.7)	Dizziness and giddiness (780.4)	Short-acting nifedipine (C08CA05)	29,762 (14.3)	Essential hypertension (401.9)
Diphenhydramine (R06AA02)	48,911 (9.9)	Dizziness and giddiness (780.4)	Diazepam (N05BA01)	24,695 (9.9)	Dizziness and giddiness (780.4)	Diazepam (N05BA01)	24,695 (11.9)	Dizziness and giddiness (780.4)
Diclofenac (M01AB05)	37,796 (8.0)	Fever (780.6)	Chlorphenamine (R06AB04)	14,187 (5.7)	Dizziness and giddiness (780.4)	Chlorphenamine (R06AB04)	14,187 (6.8)	Dizziness and giddiness (780.4)
Short-acting nifedipine (C08CA05)	29,762 (6.0)	Essential hypertension (401.9)	Chlorzoxazone (M03BB03)	12,814 (5.2)	Contusion of chest wall (922.1)	Lorazepam (N05BA06)	13,113 (6.3)	Dizziness and giddiness (780.4)
Acetylsalicylic acid >325 mg (N02BA01)	28,434 (5.8)	Fever (780.6)	Meclizine (R06AE05)	11,970 (4.8)	Dizziness and giddiness (780.4)	Ketoprofen (M01AE03)	11,794 (5.7)	Fever (780.6)
Diazepam (N05BA01)	24,695 (5.0)	Dizziness and giddiness (780.4)	Cimetidine (A02BA01)	11,627 (4.7)	Abdominal pain (789.00)	Alprazolam (N05BA12)	10,007 (4.8)	Dizziness and giddiness (780.4)
Chlorphenamine (R06AB04)	14,187 (2.9)	Dizziness and giddiness (780.4)	Pethidine (N02AB02)	9,872 (4.0)	Abdominal pain (789.00)	Pethidine (N02AB02)	9,872 (4.8)	Abdominal pain (789.00)
Scopolamine (A04AD01)	13,322 (2.7)	Other and unspecified noninfectious gastroenteritis and colitis (558.9)	Fludiazepam (N05BA17)	7,058 (2.8)	Dizziness and giddiness (780.4)	Piracetam (N06BX03)	5,058 (2.4)	Acute, but ill-defined, cerebrovascular disease (436)
Lorazepam (N05BA06)	13,113 (6.3)	Dizziness and giddiness (780.4)	Cyproheptadine (R06AX02)	4,217 (1.7)	Fever (780.6)	Zolpidem (N05CF02)	4,814 (2.3)	Dizziness and giddiness (780.4)

Dx: diagnosis; ED: emergency department; PIM: potentially inappropriate medication. The percentage of PIMs based on each set of criteria was determined by the number of PIMs/overall medications used in the ED in 2009. The percentage of each medication was determined by frequency of single drug/overall frequency of PIMs used based on each set of PIM criteria.

### Comparison of the characteristics between patients with and without at least one PIM prescription

Bivariate associations between patient, physician, hospital, and visit variables and use of PIMs are also reported (Appendix Tables 1 and 2). Women and ED patients between 65–74 years of age were more likely to be prescribed PIMs based on all three sets of criteria. For all three sets of PIM criteria, PIM users had more chronic conditions, visited more hospitals, encountered higher numbers of physicians, and were prescribed higher numbers of medications compared to patients without PIM use. Regarding visit-, physician-, and hospital-level characteristics, the associations between PIM use and associated factors were not entirely consistent based on the three sets of criteria.

Multivariate logistic regression analysis for patient-level factors revealed that women and the subjects aged between 65–74 years old had a lower risk of being prescribed PIMs in the ED based on the three sets of criteria (Table 4). Having more than three diagnoses at annual ED visits was also associated with a higher risk of PIM use. Number of hospitals visited was associated with PIM use only when the PRISCUS criteria were applied. Similarly, number of physicians encountered in the ED was associated with PIM use only when the PIM-Taiwan criteria were applied. Finally, for all three sets of criteria, the risk of being prescribed PIMs increased when patients were prescribed higher numbers of drugs at annual ED visits, especially according to the Beers Criteria.

**Table 4:** Adjusted OR for potentially inappropriate medication prescription by patient characteristics according to three sets of PIM criteria.

Characteristics	Beers Criteria		PIM-Taiwan criteria		PRISCUS criteria	
	OR	95% CI	OR	95% CI	OR	95% CI
**Patient characteristics**						
Gender						
Male	1(ref)		1(ref)		1(ref)	
Female	1.27***	1.25–1.29	1.31***	1.29–1.33	1.29***	1.27–1.31
Age (years)						
65–74	1(ref)		1(ref)		1(ref)	
75–84	0.81**	0.8–0.83	0.8***	0.79–0.82	0.91***	0.89–0.92
≥85	0.62***	0.61–0.64	0.6***	0.59–0.62	0.71***	0.69–0.72
No. of ED visits	1.26***	1.25–1.28	1.15**	1.13–1.17	1.3***	1.26–1.33
No. of hospitals	0.96***	0.93–0.98	NS		1.06***	1.04–1.09
**No. of diagnoses in ED**						
≤3/year	1(ref)		1(ref)		1(ref)	
>3/year	1.22***	1.18–1.26	1.09***	1.06–1.12	1.17***	1.14–1.2
No. of physicians in ED	NS		1.17***	1.1–1.23	NS	
**No. of medications prescribed in ED/year**						
<4	1(ref)		1(ref)		1(ref)	
4–6	3.45***	3.39–3.51	2.79***	2.73–2.84	2.53***	2.48–2.59
>6	9.18***	8.94–9.42	6.08***	5.94–6.23	5.17***	5.04–5.31

CI: confidence interval; ED: emergency department; OR: odds ratio. *P < 0.05, **P < 0.01, ***P < 0.001.

Regarding physician characteristics, the associations with PIM use differed between the three sets of criteria in the multivariate logistic regression analysis (Table 5). Physicians aged older than 40 years prescribed more PIMs to older ED patients. In contrast, older physicians prescribed fewer PIMs based on the other two sets of criteria. When physicians in the ED were certified as emergency medicine specialists, they were less likely to prescribe PIMs according to the PRISCUS criteria but were more likely to prescribe PIMs according to the Beers Criteria and PIM-Taiwan criteria.

**Table 5:** Adjusted OR for potentially inappropriate medication prescription by physician-, hospital-, and ED visit-level characteristics according to three sets of PIM criteria.

Characteristics	Beers Criteria		PIM-Taiwan criteria		PRISCUS criteria	
	OR	95% CI	OR	95% CI	OR	95% CI
**Physician characteristics**						
Gender						
Male	1(ref)		1(ref)		1(ref)	
Female	0.91***	0.88–0.93	0.86***	0.84–0.89	0.92***	0.89–0.95
Age (years)						
≤40	1(ref)		1(ref)		1(ref)	
>40	1.02**	1–1.03	0.94***	0.93–0.96	0.98**	0.97–0.99
Specialty						
Emergency medicine	1(ref)		1(ref)		1(ref)	
Non-emergency medicine	0.97***	0.96–0.98	0.92***	0.91–0.94	1.08***	1.07–1.1
**Hospital characteristics**						
Level of accreditation						
Medical center	1(ref)		1(ref)		1(ref)	
Non-medical center	1.42***	1.4–1.44	1.43***	1.4–1.45	1.32***	1.3–1.34
**Visit characteristics**						
Number of medications	1.46***	1.46–1.47	1.27***	1.27–1.27	1.25***	1.24–1.25
Duration of medication	1.03***	1.02–1.03	1.05***	1.05–1.05	1.03***	1.02–1.03

CI: confidence interval; OR: odds ratio. *P < 0.05, **P < 0.01, ***P < 0.001.

Regarding hospital- and ED visit-level characteristics, when older patients visited an ED not located in a medical center, they were more likely to be prescribed PIMs. PIM use was also associated with a higher number of medications and longer duration of prescription at each ED visit.

## Discussion

To our knowledge, our study is the first to determine the nationwide prevalence of PIM use by applying three different criteria among older ED patients to determine the difference between country-specific and non-country specific criteria. Although most of the PIM criteria were established for chronic use of medications, this study demonstrated that the prevalence of PIM use among older ED patients and at each ED visit was high, especially for the 2015 Beers Criteria. A similar number of medications were listed in the PIM-Taiwan and PRISCUS criteria, although the prevalence of PIM use was higher according to the PIM-Taiwan criteria. Male physicians, non-medical center hospital accreditation, higher number of medications, and longer duration of prescriptions were all associated with PIM use. Finally, number of medications prescribed in the ED was the most important risk factor for PIM use.

In previous studies, the reported prevalence of PIM ranged from 1.5 to 26% among older ED patients^11–14,16^. In our national database, the proportion of PIM users was larger (more than 30%), especially for the 2015 version of the Beers Criteria. The number of medications listed in these criteria may have been the major cause of the observed differences. Previous studies were based on older versions of the Beers Criteria; in contrast, the 2015 version classified more medications as PIMs and the prevalence of PIM use therefore increased. Prescribing preferences were another important factor considered. Our data showed that the leading ten PIMs were mostly prescribed for symptom relief. Moreover, the prevalence of PIM users and proportion of PIMs among all prescribed medications were higher according to the PIM-Taiwan criteria than the PRISCUS criteria. Since we decided to use the earlier established nationwide data in 2009 for this study, the effect of PIM-Taiwan criteria (published in 2012) and PRISCUS criteria (published in 2010) publication that may cause reduction of PIM could be avoided. Also, both sets of criteria have not been updated. These results confirm the advantage of using country-specific criteria that consider regional prescribing preferences and the local pharmaceutical industry.

In previous research, medication-related complications could lead to ED visits and hospitalization among older adults^21^. PIM criteria were established to discourage the use of certain drugs associated with a higher risk of complications^5^. They have been applied widely across different clinical settings, even though they were initially established to prevent the chronic use of PIMs. It has been argued that the medications used in the ED were mostly short-term or even single-dose medications. However, certain medications can cause severe adverse effects, even in a single dose. For example, ketorolac ranked as the leading PIM in our study. The adverse effects of non-steroidal anti-inflammatory drugs (NSAIDs), including a higher risk of serious cardiovascular thrombotic events, myocardial infarction, and stroke have been reported^22^. In addition, NSAIDs have been associated with a higher incidence of gastrointestinal adverse events and anaphylactoid reactions^23^. Moreover, a study conducted in a small sample reported that the efficacy of treating acute fever in the ED was similar to that of acetaminophen^24^. Our results demonstrated that PIMs should be regarded as an important issue during the prescribing process for older ED patients. It would be reasonable for ED physicians to consider avoiding PIMs and to select alternatives with less adverse effects.

Since we used large sample size of data, the factors with small association were still detected. Several factors have been identified as predictors of PIM prescription including patient (being female and 65–74 years) and hospital characteristics (non-medical center) in multivariate logistic regression analysis. These results were similar to other nationwide studies^12,16^. Bias for reported associations included study size, selection bias and other uncontrolled confounding factors. For our study, we used nationwide database (including all claim data from all regional ED) and the sample size is large with less selection bias. Our results suggested that weak association could be affected by other uncontrolled confounding factors that were not collected in the claim data such as physiological data. Among patient characteristics, the number of medications prescribed per patient was the most important factor in all three sets of criteria. If older ED patients require higher numbers of medications to treat their diseases or symptoms in the ED, drug-drug interactions and PIMs should be considered upon discharge from the ED. Therefore, a systemic strategy is needed to prevent medication-related complications which could lead to further healthcare resource utilization. A computer-based warning system has been shown to be an effective method to avoid PIM prescription in the ED^25^. Further studies are needed to verify its effectiveness at preventing medication-related complications and patient-oriented outcomes in the ED. It is interesting that the associations between PIMs and the characteristics of physicians vary between different sets of criteria. Further studies collecting detailed physician information are needed to clarify these differences.

Although it is reasonable to use PIMs for certain conditions, we could not identify all diagnoses made in the ED since the NHIRD only collected the first three diagnoses made. In Taiwan, the definite diagnosis of certain symptoms such as fever, dizziness or abdominal pain might not be made at ED and the patients were admitted for further investigation. The ICD-9 CM codes were only coding for fever, dizziness or abdominal pain which were demonstrated that leading PIM were prescribed for fever, dizziness or abdominal pain in the Table 3. Moreover, we could not use condition-specific criteria to determine the prevalence of PIM use. We anticipated that if the criteria considering chronic condition were applied to population with comprehensive data of chronic conditions, the prevalence of PIM will increase. Additionally, the claims data did contain discharge data and we therefore could not separate the medications used in the ED from those prescribed upon discharge. A further limitation is that we did not consider other adverse drug events or drug-drug interactions in this study. This is another important factor to consider in relation to the quality of prescription for older patients. Finally, the causal relationship between PIM use and patient-, physician-, and ED visit-level characteristics could not be clearly defined in this cross-sectional study. Further prospective studies will be needed to verify this relationship.

This study demonstrated that PIMs was highly prevalent among older adults visiting ED, and were not seriously considered as an issue when prescribing medications to older patients in the ED. It is essential to encourage ED physicians to consider the risk-benefit ratio when prescribing medications to older patients, especially those with polypharmacy. As iatrogenic complications are unacceptable for older patients, the avoidance of PIMs with a high risk of adverse drug effects represents an important strategy. Country-specific PIM criteria could act as a reference for clinical practice in the ED.

## Methods

### Study Design

Taiwan’s National Health Insurance Research Database (NHIRD) was developed by National Health Research Institutes. It contains a nationally representative sample of beneficiary and claim data associated with emergency care. For confidentiality control, NHRI wound not release the records for the entire population of older adults. Only claim data of older adults whose birthday is odd number were selected. Therefore, the entire database enrolled claim data of about one million older adults. This sample represented half the population of older patients reimbursed in National Health Insurance in 2009. Only those having at least one visit of emergency department were enrolled for this study. Cross-sectional study was performed to examine the frequency of PIM use among older ED patients. The Research Ethics Committee of National Taiwan University Hospital approved the project as a waved study. We confirmed that all experiments were performed in accordance with relevant guidelines and regulations.

### Study Population

We selected patients aged 65 years and older who had visited the ED at least once in 2009, regardless of their final disposition after treatment in the ED. Data collected included patient (65–74, 75–84, or ≥85 years)^26^ and physician age (≤40 or >40 years), patient and physician gender (male/female), patient diagnoses, number of ED visits, hospital accreditation, and types of oral medications prescribed in the ED. Up to three International Classification of Diseases 9^th^ edition Clinical Modification (ICD-9 CM) codes were recorded for each ED visit. Information on all medication administration in the ED included generic drug names, dose, frequency and duration. Limitations of the NHIRD included the fact that clinical data such as patients’ weight, height, blood pressure, and liver and renal function were not collected in claims data. In addition, it was difficult to define which medications were prescribed at the time of the ED visit or upon discharge from the ED. Moreover, NHIRD did not contain an exhaustive list of diagnoses for their beneficiaries, for example if one with dementia visited ED for fever, the diagnosis of dementia might not contribute to this visit and it would not be recorded for claim. Also, the definite diagnosis of certain symptoms might not be confirmed at ED, such as fever, dizziness, or abdominal pain. Therefore, we only identified PIMs independent of chronic conditions. PIMs that were considered inappropriate independent of diagnosis in the PIM-Taiwan criteria^6^ (Taiwanese), the 2015 version of the Beers Criteria^5^ (American), and the PRISCUS criteria^7^ (German) were applied to determine the frequency of PIM use and users.

### Data analysis

Analysis was conducted by two complementary perspectives: patient- and ED visit-level characteristics. All variables of interest to be examined in association with PIM were summed and presented as numbers per year. We used Kolmogorov-Smirnov test to examine the normality of continuous variables. For the ED visit-level analysis, prescription of PIMs was defined as at least one PIM based on the three sets of PIM criteria at a single ED visit. Associations of PIMS with characteristics of prescribing physicians and hospitals were investigated. For the patient-level analysis, PIMs users were defined as those who were prescribed at least one PIM in the ED. Bivariate analysis was performed using t-test or Mann-Whitney U test for continuous variables with normal or non-normal distribution, respectively. Chi-squared test for categorical variables was used to test correlations between PIMs and patient- or ED visit-level characteristics. Stepwise multivariate logistic regression models were used to identify the correlates of having at least one PIM at the patient and ED visit level after adjusting for potential confounders. All tests conducted were two-tailed, and significance was set at p < 0.05. The top ten PIMs from each set of PIM criteria were ranked by calculating the percentage of PIMs divided by total number of medications prescribed for older patients having at least one ED visit in 2009. Data were analyzed using SAS for Windows version 9.3 (SAS Institute Inc., Cary, NC, USA).

### Data availability

The data that support the findings of this study were obtained from National Health Research Institutes (NHRI) of the Ministry of Health and Welfare in Taiwan. There are restrictions for the availability of these data, which should be used under license, and the database was not publicly available for duplication.

## Electronic supplementary material

Appendix tables

## References

[1] Aminzadeh F, Dalziel WB (2002). Older adults in the emergency department: a systematic review of patterns of use, adverse outcomes, and effectiveness of interventions. Annals of emergency medicine.

[2] Huang JA, Weng RH, Tsai WC, Hu WH, Yang DY (2003). Analysis of emergency department utilization by elderly patients under National Health Insurance. The Kaohsiung journal of medical sciences.

[3] Wu TY, Majeed A, Kuo KN (2010). An overview of the healthcare system in Taiwan. London journal of primary care.

[4] Otero Lopez MJ, Bajo Bajo A, Maderuelo Fernandez JA, Dominguez-Gil Hurle A (1999). Preventable adverse drug effects at an emergency department. Revista clinica espanola.

[5] By the American Geriatrics Society Beers Criteria Update Expert, P (2015). American Geriatrics Society 2015 Updated Beers Criteria for Potentially Inappropriate Medication Use in Older Adults. Journal of the American Geriatrics Society.

[6] Chang CB (2012). Using published criteria to develop a list of potentially inappropriate medications for elderly patients in Taiwan. Pharmacoepidemiology and drug safety.

[7] Holt S, Schmiedl S, Thurmann PA (2010). Potentially inappropriate medications in the elderly: the PRISCUS list. Deutsches Arzteblatt international.

[8] Morgan SG (2016). Frequency and cost of potentially inappropriate prescribing for older adults: a cross-sectional study. CMAJ open.

[9] Shah KN, Joshi HM, Christian RP, Patel KP, Malhotra SD (2016). Prevalence of potentially inappropriate medications and prescription cost analysis among older cardiac patients in an outpatient department of a tertiary care hospital in India. Journal of basic and clinical pharmacy.

[10] Al-Omar HA, Al-Sultan MS, Abu-Auda HS (2013). Prescribing of potentially inappropriate medications among the elderly population in an ambulatory care setting in a Saudi military hospital: trend and cost. Geriatrics & gerontology international.

[11] Heininger-Rothbucher D (2003). Problematic drugs in elderly patients presenting to a European emergency room. European journal of internal medicine.

[12] Caterino JM, Emond JA, Camargo CA (2004). Inappropriate medication administration to the acutely ill elderly: a nationwide emergency department study, 1992–2000. Journal of the American Geriatrics Society.

[13] Hustey FM, Wallis N, Miller J (2007). Inappropriate prescribing in an older ED population. The American journal of emergency medicine.

[14] Meurer WJ (2010). Potentially inappropriate medication utilization in the emergency department visits by older adults: analysis from a nationally representative sample. Academic emergency medicine: official journal of the Society for Academic Emergency Medicine.

[15] Wallace E, McDowell R, Bennett K, Fahey T, Smith SM (2017). Impact of Potentially Inappropriate Prescribing on Adverse Drug Events, Health Related Quality of Life and Emergency Hospital Attendance in Older People Attending General Practice: A Prospective Cohort Study. The journals of gerontology. Series A, Biological sciences and medical sciences.

[16] Chen YC (2009). Potentially inappropriate medication for emergency department visits by elderly patients in Taiwan. Pharmacoepidemiology and drug safety.

[17] Hustey FM (2008). Beers criteria and the ED: an adequate standard for inappropriate prescribing?. The American journal of emergency medicine.

[18] West LM, Cordina M, Cunningham S (2012). Clinical pharmacist evaluation of medication inappropriateness in the emergency department of a teaching hospital in Malta. Pharmacy practice.

[19] Farfel JM (2010). Adverse drug events leading to emergency department visits in elderly: the role of inappropriate prescription. Einstein.

[20] Nixdorff N (2008). Potentially inappropriate medications and adverse drug effects in elders in the ED. The American journal of emergency medicine.

[21] Hohl CM, Dankoff J, Colacone A, Afilalo M (2001). Polypharmacy, adverse drug-related events, and potential adverse drug interactions in elderly patients presenting to an emergency department. Annals of emergency medicine.

[22] Peterson, K. *et al*. *Drug Class Review: Nonsteroidal Antiinflammatory Drugs (NSAIDs): Final Update 4 Report [Internet]. Portland (OR): Oregon Health & Science University Appendix B, Black Box Warnings of Included Drugs*., Available from: https://www.ncbi.nlm.nih.gov/books/NBK53952/ (2010 Nov). 21542548

[23] Chung HS, Kim ES, You YJ, Park CS (2010). Anaphylactoid reaction after injection of ketorolac in a loading dose for patient-controlled analgesia -A case report. Korean journal of anesthesiology.

[24] Houry D, Ernst A, Weiss S, Ledbetter M (1999). Ketorolac versus acetaminophen for treatment of acute fever in the emergency department. Southern medical journal.

[25] Terrell KM (2009). Computerized decision support to reduce potentially inappropriate prescribing to older emergency department patients: a randomized, controlled trial. Journal of the American Geriatrics Society.

[26] Loraine, A. West, S. C., Daniel, G. & Wan, He. (U.S. Census Bureau) 23–212 (U.S. Government Printing Office, Washington, DC, 2014).

