# Supplementary material for: Prescription of potentially inappropriate medication to older patients presenting to the emergency department: a nationally representative population study
Source: Sci Rep. 2018 Aug 6;8:11727. doi: 10.1038/s41598-018-30184-4 (PMC6078949; doi:10.1038/s41598-018-30184-4)
Supplement: Supplementary file 1 — Appendix tables [file 41598_2018_30184_MOESM1_ESM.doc]

# Title of Manuscript

**Prescription of potentially inappropriate medication to older patients presenting to the emergency department: a nationally representative population study**

**Author List: Chirn-Bin Chang,^1,2,3^ Hsiu-Yun Lai,^4^ Shinn-Jang Hwang,^5,6^ Shu-Yu Yang,^7,8^ Ru-Shu Wu,^9^ Hsing-Cheng Liu,^8,10^ Ding-Cheng Chan* ^2,3,11^**

Appendix Table 1. Comparison of the characteristics between patients with and without at least one PIM prescription (N = 313,733)

|  | Beers Criteria |  | *p-*value | PIM-Taiwan criteria |  | *p-*value | PRISCUS criteria |  | *p-*value |
| --- | --- | --- | --- | --- | --- | --- | --- | --- | --- |
|  | With PIM  n = 199,871 | Without PIM  n = 113,862 |  | With PIM  n = 132,186 | Without PIM  n = 181,547 |  | With PIM  n = 101,646 | Without PIM  n = 212,087 |  |
| Age (years)  (mean ± SD)  65–74  75–84  ≥ 85 | 76.5±7.3  91,144 (65.3)  81,598 (63.4)  27,140 (59.6) | 77.1±7.5  48,346 (34.7)  47,070 (36.6)  18,435 (40.5) | <0.001  <0.001 | 76.3±7.2  61,829 (44.3)  53,588 (41.7)  16,769 (36.8) | 77.1±7.5  77,661 (55.7)  75,080 (58.4)  28,806 (63.2) | <0.001  <0.001 | 76.5±7.1  46,147 (33.1)  42,272 (32.9)  13,227 (29.0) | 76.9±7.5  93,343 (66.9)  86,396 (67.2)  32,348 (71.0) | <0.001  <0.001 |
| Men, N (%) | 98,470 (49.3) | 60,982 (53.6) | <0.001 | 63,182 (47.8) | 96,270 (53.0) | <0.001 | 48,155 (47.4) | 111,297 (52.5) | <0.001 |
| No. of chronic diseases  (mean ± SD) | 3.5±2.5 | 2.3±1.4 | <0.001 | 3.7±2.7 | 2.6±1.7 | <0.001 | 3.8±2.8 | 2.7±1.8 | <0.001 |
| No. of annual ER visits | 2.2 ±2.5 | 1.3±0.8 | <0.001 | 2.4±2.8 | 1.5±1.1 | <0.001 | 2.5±3.1 | 1.5±1.1 | <0.001 |
| No. of hospitals | 1.2±0.5 | 1.1±0.3 | <0.001 | 1.3±0.6 | 1.1±0.4 | <0.001 | 1.3±0.6 | 1.1±0.4 | <0.001 |
| No. of physicians | 1.9±1.4 | 1.3±0.6 | <0.001 | 2.1±1.6 | 1.4±0.8 | <0.001 | 2.2±1.7 | 1.4±0.9 | <0.001 |
| No. of medications prescribed in ER | 7.6±5.4 | 3.8±2.6 | <0.001 | 8.3±5.9 | 4.7±3.4 | <0.001 | 8.7±6.2 | 5.0±3.6 | <0.001 |

ER: emergency room; PIM: potentially inappropriate medication

Appendix Table 2. Comparison of visit-, physician-, and hospital-level characteristics between ED visits with and without at least one PIM prescription (N = 579,796)

|  | **Beers Criteria** |  | *p-*value | **PIM-Taiwan criteria** |  | *p-*value | **PRISCUS criteria** |  | *p-*value |
| --- | --- | --- | --- | --- | --- | --- | --- | --- | --- |
|  | With PIM  n = 309,800 | Without PIM  n = 269,996 |  | With PIM  n = 186,673 | Without PIM  n = 393,123 |  | With PIM  n = 141,576 | Without PIM  n = 438,220 |  |
| **Visit characteristics** |  |  |  |  |  |  |  |  |  |
| No. of medications prescribed in ED  (mean ± SD) | 4.8±2.5 | 3.2±2 | <0.001 | 5±2.5 | 3.6±2.3 | <0.001 | 5.1±2.7 | 3.7±2.2 | <0.001 |
| Duration of medication (days)  (mean ± SD) | 2.5±1.6 | 2.3±1.7 | <0.001 | 2.6±1.6 | 2.3±1.7 | <0.001 | 2.6±1.6 | 2.4±1.7 | <0.001 |
| Cost of medication (TWD)  (mean ± SD) | 376.8±1524 | 308.9±2713 |  | 348.2±1555.2 | 343.7±2395.4 | <0.001 | 341.8±1693.2 | 346±2291.5 |  |
| **Physician characteristics** |  |  |  |  |  |  |  |  |  |
| MD age (years)  (mean ± SD) | 40.9±8.1 | 40.8±8.3 | <0.001 | 40.7±8 | 40.9±8.3 | <0.001 | 40.9±8.2 | 40.8±8.2 | <0.001 |
| MD age ≤ 40 | 158,431 (52.9) | 141,171 (47.1) | <0.001 | 96,832 (32.3) | 202,770 (67.7) | 0.04 | 72,287 (24.1) | 227,315 (75.9) | <0.001 |
| MD age > 40 | 151,369 (54) | 128,825 (46) |  | 89,841 (32.1) | 190,353 (67.9) |  | 69,289 (24.7) | 210,905 (75.3) |  |
| MD gender |  |  | <0.001 |  |  | <0.001 |  |  |  |
| Female | 20,640 (51.8) | 19,186 (48.2) |  | 11,844 (29.7) | 27,982 (70.3) |  | 9,369 (23.5) | 30,457 (76.5) |  |
| Male | 288,194 (53.6) | 249,778 (46.4) |  | 174,328 (32.4) | 363,644 (67.6) |  | 131,746 (24.5) | 406,226 (75.5) |  |
| MD specialty |  |  | <0.001 |  |  | <0.001 |  |  | <0.001 |
| Emergency medicine | 158,474 (53.9) | 135,538 (46.1) |  | 97,874 (33.3) | 196,138 (66.7) |  | 70,906 (24.1) | 223,106 (75.9) |  |
| Non-emergency medicine | 117,322 (52.7) | 105,512 (47.4) |  | 68,991 (31) | 153,843 (69) |  | 55,681 (25) | 167,153 (75) |  |
| **Hospital characteristics** |  |  |  |  |  |  |  |  |  |
| Hospital accreditation |  |  | <0.001 |  |  | <0.001 |  |  | <0.001 |
| Academic medical center | 82,639 (50.6) | 80,837 (49.5) |  | 48,194 (29.5) | 115,282 (70.5) |  | 37,683 (23.1) | 125,793 (77) |  |
| Non-medical center | 227,161 (54.6) | 189,159 (45.4) |  | 138,479 (33.3) | 277,841 (66.7) |  | 103,893 (25) | 312,427 (75) |  |

ED: emergency department; MD: Doctor of Medicine (physician); PIM: potentially inappropriate medication; TWD: New Taiwan Dollar, equivalent to 0.03 USD at time of publication
